# The Assemblage and Dismantling of Access Barriers in Administrative Bureaucracies: Constructing the Problem of Diversity in the German Welfare State

**DOI:** 10.1007/s11133-023-09555-5

**Published:** 2024-01-04

**Authors:** Martin Petzke

**Affiliations:** Bielefeld University, Universitätsstr. 25, 33615 Bielefeld, Germany

**Keywords:** Actor-network theory, Ontology, Performativity, Diversity management, Social mobility

## Abstract

The article extends the literature on the construction of “diversity management” by personnel managers in corporate America. Such research has highlighted that Human Resource (HR) specialists draw heavily on social-scientific thinking in implementing various remedies against discrimination. However, it has paid less attention to how such esoteric views of reality, comprising such “things” as “structural barriers” impeding occupational advancement and “diversity sensitivity,” have been successfully established as a self-evident reality in the workplace. In order to more thoroughly investigate how the world of diversity management is established outside the circle of academic specialists, the article employs perspectives from science and technology studies on the ways in which sociotechnical assemblages, i.e., networks of human actors and material devices, enact scientific ontologies. It applies such perspectives to a German case of diversity management, a program of “intercultural opening” that seeks to make bureaucracies of the welfare state more accessible to immigrants. The article delineates the specific ontology behind this version of diversity management, rooted in sociological perspectives on social mobility, and explores the various techniques and instruments through which officers of intercultural opening establish this ontology as a visible reality in municipal administrations.

## Introduction

Sociological perspectives on the implementation of antidiscrimination policies in the workplace have stressed the role of particular professions in translating vague legislation and court rulings into concrete directives for organizations. Skrentny (1996) has highlighted how an “administrative pragmatism” led bureaucrats in government agencies to reimagine the originally color-blind policy of affirmative action as a race-conscious policy of numerical targets that granted administrators a more proactive role in enforcing civil rights and the means to demonstrate success. Dobbin and Kelly (2007) have explored how personnel managers overstated risks of sexual harassment litigation and exaggerated the extent to which procedures from their own professional tool kit such as grievance procedures and sensitivity training provided legal protection (see also Edelmann et al. 1992). Similarly, they have shown that when the federal government cut back on affirmative action enforcement, HR specialists and equal opportunity officers recast antidiscrimination measures as “diversity management,” promoting the economic benefits of a diverse workforce (Kelly and Dobbin 1998). The construction of business arguments salvaged old procedures but also brought new measures such as the “culture audit” to the mix, where consultants seek to identify institutional barriers to diversity and minority advancement in the organization.

Such work has provided invaluable insights into the ways in which professional interests have shaped our present understanding of equal opportunity and diversity. I build on these insights as I further explore an aspect which is highlighted but somewhat underdeveloped in the literature above. In implementing antidiscrimination measures, personnel managers draw substantially from social-scientific expertise. In fact, as Dobbin (2002) points out, the development of antidiscrimination measures closely mirrored the changing paradigms in social-scientific thinking. As institutional explanations for occupational attainment and discrimination succeeded behavioral ones, and cognitive explanations succeeded institutional ones, personnel managers tuned their arsenal of preventive measures accordingly (Dobbin 2002; 2009).

However, social-scientific perspectives on such “things” as “unintentional discrimination,” “institutional structures,” or “unconscious categorizations” are anything but self-evident. Neither are the antidiscrimination measures derived from such elaborate theoretical models. This raises an important question: how could personnel specialists bring employers and employees in mundane organizations to look at the world in such unusual and counterintuitive terms? The literature above mostly focuses on the ways in which HR managers successfully convince employers of the risk of litigation and the economic value of diversity. What follows from that success, however, can in many ways be seen as a much more ambitious project: the installment of a social ontology populated by such “things” as “structural barriers,” “cultural diversity,” and “diversity sensitivity.” This article turns to this subtle yet fundamental project of worldmaking by a subgroup of professionals in various organizations, which is implied yet largely unexplored in the work above. It sheds light on some of the more finespun processes through which a specialist ontology of “diversity” is transforming a broader “social imaginary” (see Vertovec 2012).

In order to investigate the ways in which a model of the social world derived from the social sciences is established as a social reality of the workplace, I turn to the literature of science and technology studies. Specifically, I draw on the work of Bruno Latour, Michel Callon, and Donald MacKenzie that attends to the ways in which scientific theories intervene in and shape social realities via sociotechnical assemblages and “actor-networks” of humans and material devices (Callon 1984; 1998; 2007; Callon and Latour 1981; Latour 1987; 1993; 2005; MacKenzie 2006; MacKenzie and Millo 2003). In his seminal study on the Pasteurization of France, Latour (1993) has argued that the reality of the infectious microbe was established through extending a network of Pasteurians, petri-dishes, microscopes, means of epidemiological observation, and sterile utensils. In essence, the theory of the microbe “worked” only under laboratory conditions. Thus, in order for the microbe to become an established and recognized element of the social world, the sociotechnical arrangements of the laboratory had to move into the world, effectively turning it into a laboratory on a macroscopic scale.

Callon (1998; 2007) and MacKenzie (2006; see also MacKenzie and Millo 2003) have more recently brought such perspectives to bear on the role of economists in constructing the reality of economic markets. They highlight economics’ “performative” effects as they show how conceptual frames, calculative procedures, and technical instruments derived from economic models establish realities and elicit behavior in line with the predictions of such theories. In the case of the financial derivatives exchange market, for instance, concepts from an economic model of option pricing increasingly governed the way traders thought and talked about their trade, and material devices such as pricing sheets and computer applications continually fed calculations of the model into the trading process as realities to be reckoned with (MacKenzie and Millo 2003). Thus, rather than objectively registering a state of the world, economic theories quite conversely remake the world in their own image.

I argue that utilizing such perspectives can help us gain analytical leverage on the work of “ontological worldmaking” (Woolgar and Lezaun 2013, 322) involved in establishing organizational practices of diversity management. After all, much of diversity management builds on somewhat esoteric social-scientific notions about the social world that are unlikely to be immediately intuitive to those outside of academic circles. In consequence, the construction of the problem of diversity, or, more precisely, the problem of a lack thereof, in many ways involves bringing people to treat as objective realities various entities that previously have not been part of the world that they themselves inhabit, such as barriers impeding access or occupational advancement, and faculties like diversity sensitivity. It means bringing their world in alignment with the world of diversity management and the social-scientific ontology on which it rests. Science and technology studies can sensitize us to the ways in which such an alignment relies on sociotechnical assemblages that evoke specific social-scientific (and psychological) realities.

In order to explore the process through which professionals in organizations install the particular ontology of diversity management, or more specifically, how they construct a world that would necessitate diversity management, I look at a program promoting an “intercultural opening” of administrative bureaucracies in Germany.^1^ Within recent decades, administrative agencies on all levels of government have latched on to what is essentially corporate diversity management under a different name. The concept of an “intercultural opening” of public bureaucracies of the German welfare state surfaced in the 1990s, propelled by voices that advocated for a reckoning with the idea that Germany was an immigration nation, and immigrants were here to stay (see Barwig and Hinz-Rommel 1995). It calls for adapting bureaucratic structures to the realities of an increasingly diverse population by way of making them more accessible and maneuverable for immigrants. Such invocations of an “intercultural opening” have gained even greater currency since the 2000s and form a substantial component of the National Integration Plan of 2007, a programmatic statement of Germany’s new integration policy that has been ushered in by the Social Democrat/Green Party coalition of 1998 and pushed further by the various administrations under Chancellor Angela Merkel. In the following, I explore such a program sponsored by the department for immigrant integration of a large territorial state in Germany. The program employs officers in all municipalities within the state who are responsible for interculturally opening the municipal bureaucracy. As ambassadors for intercultural opening, these officers occupy analogous positions to the diversity managers of corporate America.

Looking at a German case to explore the ways in which an ontology of intercultural diversity, access barriers, and diversity sensitivity, or “intercultural competence,” is installed in organizations bears several advantages. For one, in Germany issues of race, cultural diversity, and equal opportunity in the workplace have garnered far less political attention and public significance as compared to the US, and they have only recently gained traction (Joppke 2007; Schiller et al. 2020; Schönwälder and Triadafilopoulos 2016). Whereas the public discourse in the US today is saturated with social-scientific ideas and concepts pertaining to diversity, race, and cultural difference, such perspectives are not as self-evident or even familiar to people in the German workplace.^2^ This holds even truer for public bureaucracies where ideas on diversity do not circulate internationally to the extent that they do in the corporate world. German administrations thus constitute a particularly informative case to observe the actual work involved in “enculturating” other people into the world of diversity management imbued with social-scientific perspectives on occupational attainment, social mobility, and systemic discrimination.

In investigating a diversity program implemented in a western territorial state of Germany, the article highlights how state-employed officers for “intercultural opening” try to impose on local administrators in municipal administrations a specific ontology comprising such “things” as the underrepresentation of “persons with a migration background” in the bureaucracy, “access barriers,” and “intercultural competence” as a tool of dismantling such barriers. Drawing on perspectives from science and technology studies, I argue that we find here a process analogous to the one explored in Latour’s (1993) study on Louis Pasteur. Just as the “Pasteurization of France” involved stabilizing an ontology of the microbe through a network of instruments and human actors such as petri-dishes, microscopes, manuals, and Pasteurians, so does the enactment of sociological perspectives on social mobility in the name of “intercultural opening”—or what one may analogously call the “interculturalization” of German bureaucracies—rely on a network of statistical instruments, surveys, guidelines, and ambassadors for intercultural opening.

The contributions of this article are twofold. First, it provides a complement to the explanations of how organizational practices of diversity management and antidiscrimination have diffused into organizations. In attending to professional networks and the ways in which affirmative action measures have been retheorized as diversity management, Kelly and Dobbin (1998) already complicate an overly simple story of diffusion, which is a central target of Latour’s theoretical intervention (e.g., Latour 1987, 132–143). In many ways, Kelly and Dobbin’s account already resonates with perspectives of actor-network theory. It is a “Machiavellian” (Latour 1987, 129) view of professionals enlisting employers in their agenda of diversity management by appealing to business arguments in what may be deemed an act of “translation” (Latour 1987, 132; see also Callon 1981). This article completes this picture by adding the “missing masses” (Latour 1992) to the equation (see also Rodríguez-Muñez 2016): artifacts and material devices that are pivotal in evoking and making obvious the “realities” of diversity management.

Second, the article contributes to a literature on “performativity” which has brought the broader concerns of science and technology studies to the object of the social sciences and the question of how they themselves are implicated in the constitution of social realities. However, such investigations have so far been limited to the realm of economics and have mostly refrained from posing a similar question to sociology itself (but see Law 2009; Rutherford 2017; also Osborne and Rose 1999). A great potential and indeed early program specifically of actor-network theory thus remains largely unexplored: i.e., investigating how actors and instruments equipped with *sociological* “ontologies” of the world engender the realities conveyed by such conceptions of the social (see Callon and Latour 1981). Here, actor-network theory both continues and extends the ethnomethodological program of examining how “social facts” receive their objectivity and obdurateness in the first place (Garfinkel 2002; Zimmerman and Pollner 1970). Both paradigms break with what they see as shared plausibility structures between “professional” and “lay” sociologists in order to analyze how such assumptions about the social world are generated and maintained. Actor-network theory, however, goes beyond ethnomethodology in emphasizing the constitutive role of science and materiality in the process (on this juxtaposition see Callon and Latour 1981). Yet, notwithstanding the study of “looping” effects of classification systems (Hacking 2002; 2007) or the “reactivity” of social- scientific indicators (Espeland and Sauder 2007), actual empirical accounts of how sociological imaginaries of the world and its composition are enacted and implemented so as to produce that very world remain sparse. This article addresses this lacuna in highlighting how a pervasive ontology of the sociology of occupational attainment and social mobility, which reifies structural barriers that block otherwise universal trajectories for certain individuals, is enacted and made “real” by officers for “intercultural opening” in municipal administrations.

In the following section, I briefly describe the case of integration policy in Germany before turning to the discussion of my data and methodology. I then delineate how the discipline of sociology arrived at an ontology of “structural barriers” and how this has carried over into research on social mobility and occupational attainment. As I show in the following section, it is this ontology of access barriers in need of dismantling that also informs the outlook on immigrant integration and intercultural opening prevalent in German bureaucracies. In the subsequent section, I follow the officers for intercultural opening in their attempts to install this ontology of integration in municipal administrations, highlighting the practices and instruments involved in making a world of diversity management. I conclude with a discussion of some larger implications of this study.

## Integration Policy and Intercultural Opening in Germany 

The turn of the century witnessed a break with established notions of immigration in Germany. With the rise to power of a Social-Democrats/Green Party coalition, Germany officially acknowledged its status as an immigration nation after decades of denial, and soon pushed through immigration and citizenship reform. “Integration,” i.e., the social incorporation of immigrants and their descendants, previously an anathema, now increasingly gained traction as a political issue. It was in this climate that many German states and municipalities increasingly created integration offices as administrative units seeking to support immigrants in becoming settled and established in the German society (see also de Graauw and Vermeulen 2016).

In instituting integration offices and their specific problem perspective on immigrant integration since the 2000s, administrators on the municipal, state, and the federal level availed themselves of much sociological expertise, and even enlisted migration research institutes and sociologists as consultants. On all levels, many integration departments have installed so called integration monitoring instruments, i.e., compilations of official statistics that seek to operationalize and assess the state of integration among the immigrant population. These monitors comprise such indicators as level of education, employment, income, health, civic engagement, and political involvement. In order to gain a measure of integration, the reports compare the segment of “persons with a migration background,” or first- to third-generation immigrants, with “persons without a migration background” on these parameters, highlighting discrepancies or, from the point of view of institutional sectors, underrepresentation as an integration deficit. In line with much of social inequality research and the sociology of immigrant integration (see Drouhout and Nee 2019), policies oriented by these statistics seek to close the gap between both population segments and steer towards equal representation in all institutional sectors, or, in other words, to eliminate the systematic effect of a migration history on life trajectories.

One specific agenda resonating with such perspectives of underrepresentation and gaps is that of an “intercultural opening” of public bureaucracies, surfacing in the 1990s in Germany among early proponents of immigration reform. It aims to make German bureaucracies more accessible for clients with a history of immigration by raising awareness for cultural diversity (see Barwig and Hinz-Rommel 1995).

“Intercultural opening” is a somewhat vague and protean concept, a quality it shares with the signifier “diversity” (see Vertovec 2012). Even early proponents of the initiative now concede that use of this formula in integration policy discourse has become somewhat “inflationary” and “taken-for-granted” (Schröer 2007, 8). What seems to be widely accepted is that two core components of intercultural opening are a staffing of administrations with a representative share of persons with a migration background as well as trainings in “intercultural competence.” Migrants’ access to administrative services is assumed to be facilitated by encountering persons with a migration background among the administrators. In addition, trainings in “intercultural competence” among the entire staff are thought to further contribute to the levelling of “access barriers for migrants in the administration and in utilizing administrative services and offers” (Federal Government of Germany 2007, 112).

The initiative of intercultural opening has obvious affinities with “diversity management” of corporate America. Administrators in the public sector in Germany, however, consciously chose not to adopt this term, which they see as primarily emphasizing economic benefits of diversity. Such branding strategies notwithstanding, we find here similar constructions of the problem of minority representation and similar tools offered by those claiming jurisdiction for it, such as sensitivity trainings.

This article looks at a specific implementation of “intercultural opening” in what shall here be referred to as the state of Westlanden, a territorial state in the western part of Germany with a comparatively large population with a migration background (approximately 30%). Westlanden launched a statewide program designed to promote processes of intercultural opening in municipal administrations. All municipalities in the state have enrolled and now employ an officer in their local administration who carries the responsibility of pushing and implementing an agenda of interculturally opening local administrative structures. Many of these officers have an academic background in one of the social sciences, such as sociology, cultural studies, anthropology, or social work, though this is not an explicit requirement of the program.

As I will elaborate in this article, this program is very much informed by the sociological ontology of social mobility research that generally characterizes bureaucratic perspectives on immigrant integration in Germany. It thus serves as a pertinent case to explore how the sociological model of society implicit in “diversity management” is enacted and “made real” in the world outside academia.

## Data and Methods

This article is part of a larger study that investigates the ways in which sociological expertise and the statistical quantification of integration influences the work of integration officials in Germany. Relevant data for this article come from the federal level, the state of Westlanden, as well as the municipalities enrolled in the program of intercultural opening in Westlanden. The state of Westlanden was chosen for its comparatively long-standing and extensive engagement in integration policy as well as in the quantification of integration. Its ambitious program of a statewide “intercultural opening” is indicative of both: (1) it promotes the inclusion of immigrants in bureaucracies, and (2) it promotes municipal reports that quantitatively monitor immigrant integration.

In order to ascertain the specific ontology of integration prevalent in German integration departments, I analyzed integration reports, policy documents, and program reports on the federal, state, and municipal level as well as the course materials for a course on intercultural competence from a nationally renowned institute for intercultural trainings. I also conducted ethnographic observation of official integration conferences and conferences on the general topic of “intercultural opening,” of administrative working groups and executive committees within integration departments, of inter-municipal network meetings among integration officers, and of sessions of political committees for integration.

I turned to secondary sources on the history of sociology to reconstruct the social-scientific ontology that evidently informs the ontology of integration enacted by integration officers and ambassadors for intercultural opening. I also draw on sociological and historical work on minority politics and social policy to demonstrate how such ontologies have percolated into the public sector in the US and corroborate these findings with a Google Ngram analysis.

In order to explore how such ontologies are enacted in the wider bureaucracy outside of the integration departments in Germany, I joined two retreats (one lasting two days, the other one day) and a full-day conference of the collective of officers in the program of intercultural opening, where strategies of intercultural opening and experiences in the municipal administrations were discussed. I further conducted 25 in-depth interviews as well as 14 informal interviews with the officers for intercultural opening and with federal, state, and municipal administrators between June 2017 and December 2017. Documents, field notes, and transcripts of interviews were coded following an abductive, iterative approach, informed by the central tenets of grounded theory (Charmaz 2006) but in close dialogue with the theoretical repertoire of sociology (Timmermans and Tavory 2012), and using atlas.ti. All interview subjects were informed that the interest of the study lies in the ways in which understandings of immigrant integration take shape in bureaucracies. Evidence cited in this paper has been translated from German into English.

## The Sociological Ontology of Society in Social Mobility Research

The ontology which informs the policy discourse on integration and intercultural opening in Germany has its roots, as I argue, in sociology’s construction of society and social structure, especially in the context of social mobility research. In many ways, sociology was intricately involved in inventing “the social” or “society” as an object of rational investigation and planned intervention in the nineteenth century (Steinmetz 1993). Social affairs have of course always been a matter of intellectual engagement and general observation in any human collective. However, the modern concept of “society” and the attendant reification of the social as a realm of autonomous forces and dynamics is a relatively recent product. It was born out of the breakdown of the feudal order in Europe and with it the pervasive deregulation of social action (see Tenbruck 1981; see also Nisbet 1943; Wagner 2000). Order was no longer perceived as fixed by tradition or sacred statute but open to change, even to experimentation, with potentially cumulative, unanticipated, and incalculable effects. As a newly emerging discipline, sociology cast this perception into its inaugural concepts of “Gemeinschaft” vs. “Gesellschaft” (Tönnies) or “mechanical” vs. “organic solidarity” (Durkheim) and laid claim to investigating social forces and their laws, akin to natural forces in the domain of the natural sciences.

Perhaps no other sociological tradition exemplifies this reification of “the social” more pronouncedly than the French one, with Emile Durkheim as its key exponent (Tenbruck 1981). Not coincidentally, France exhibited arguably the most drastic and sudden rupture between its feudal past and modern order. It is in Durkheim’s work that the prototypical sociological ontology is most distinctly fleshed out. His “chosisme” envisions “faits sociaux” or “social facts” as exterior entities, palpably and autonomously exerting constraints and acting upon the individual, making themselves felt like forces of a natural kind. But on the other side of the Rhine, too, Max Weber’s notion of “life chances,” and his frequent use of similar compounds with the word “chance,” likewise point to something exterior, autonomous, and unyielding, to “structurally anchored probabilities of the occurrence of certain events” (Dahrendorf 1979, 67).

The idea of “society” as first and foremost a constraining “social structure” pervades sociology to this day, and Robert K. Merton played a pivotal role in consolidating it further (see Coser 1975; Merton 1995). His seminal paper on “Social Structure and Anomie” elaborated a sociological ontology of things and forces that has extended far beyond deviancy research, permeating deep into research on social mobility. Merton’s argument that some people seek alternative means of social advancement given their lack of opportunities ushered in an understanding of the social world populated by “barriers,” “obstacles”, “blockage of access” or “open doors,” and, not least, individuals with very similar aspirations (see Merton 1968, 216–17, 238).

The expressly probabilistic dimension of Merton’s ideas in itself already exhibited affinities with the quantitative outlook of social mobility research that gained ground in the following decades. But causal empiricism, as it would come to dominate much of social science, mirrored a classical Durkheimian ontology in many other ways as well, though it would arrive via different routes (Abbott 2001). The “watershed” came with the introduction of path analysis to sociology, which established a concept of causality in quantitative research resonating “with both the quality of emergentism and the character of forcing or determination, both of which are present in Durkheim” (Abbott 2001, 110–13). A characteristic and seminal example in this regard is Blau and Duncan’s (1967) classic work on the “American Occupational Structure,” which identifies the structural forces that shape the life trajectories of Americans. Peter M. Blau, of course, had been a student of Robert K. Merton at Columbia.

Today’s research on inequality and mobility is replete with Merton’s metaphors of access barriers and obstacles. It also shares the often-implicit assumption of a more or less equal distribution of individual aspirations throughout all segments of society (Abbott 2016). The premise of such an ontology is that all facets or institutional sectors of social life should in principle exhibit a distribution of specific (ascriptive) social attributes proportionate to the distribution in the general population. It rests on the tacit model of a “bingo game structure” (Collins 1984, 336) or thermodynamic diffusion (Abbott 2016, 241), in which individuals, regardless of group affiliation, are expected to diffuse equally through social space if not otherwise impeded. Thus, such statistical assumptions of a random or uniform distribution of aspirations of social advancement naturally dovetail with an ontology of “barriers”: skewed distributions and imbalances will inevitably be attributed to “exterior” impediments.

**Figure 1:**
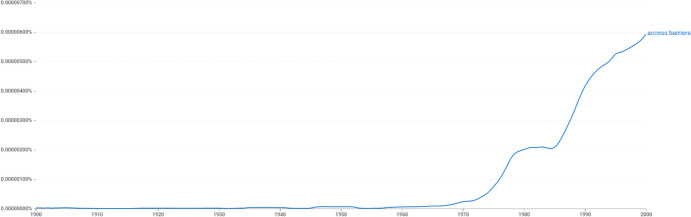
Google Ngram of “access barriers” for all English books

Such semantics of “barriers” percolated deep into minority politics and social policy as administrators in official agencies proactively began using statistical analyses in constructing the numerical underrepresentation of minorities as a public problem (see Skrentny 1996; 2002). Landmark US Supreme Court decisions such as *Griggs vs. Duke Power Co.* of 1971, which outlawed employment tests with a “disparate impact” on minorities, were central in establishing ideas of structural discrimination outside of academia (Dobbin and Sutton 1998). A Google NGram analysis, which measures the proportion of references to a phrase among a large corpus of English texts, shows that the term “access barriers” proliferates rapidly in the wake of these developments in the 1970s (see Fig. 1). Comments by Mary Keyserling, the director of the Labor Department’s Women’s Bureau, in an interview on the promotion of minority rights for women in October 1968 exemplify how the world was now seen through a lens of trajectories and blockages:

The emphasis was very largely on correcting underutilization. It turned from mere declaration of rights to the actual *opening of the doors* of opportunity. There was still much door-opening to be done, and it was done through law. But it was also— ‘Let’s find out why people aren’t going through those doors and if there are *barriers* that are keeping them from realizing the opportunities now released through change in law. Let’s meet those problems specifically. (Quoted in Skrentny 2002, 131; my emphases)

Indeed, by the 1960s not only the typical quantitative measurements of underrepresentation, but, as O’Connor (2001, 128–129) points out, also Merton’s theory of differential opportunity structures had found its way into “interlocking social science, philanthropic, and federal agency networks” of poverty knowledge, with Merton’s student Richard Cloward and his collaborator Lloyd Ohlin playing a pivotal role.

As I shall detail in the following, integration policy in Germany subscribes wholeheartedly to this particular sociological imagination of society.

## The Sociological Ontology of Integration Policy in Germany

German bureaucrats availed themselves of much sociological expertise in implementing an administrative jurisdiction for the problem of integration after Germany’s turn to integration policy in the early 2000s. It is thus no surprise that the integration reports clearly subscribe to the implicit notion of a “bingo game structure” (Collins 1984, 336) of the social universe so typical of quantitative inequality research. This manifests itself in pervasive references to “chance” in the reports: educational chances, employment chances, integration chances, life chances, social advancement chances, participation chances, self-actualization chances, development chances, job market chances, and career chances are just some of the compounds used in this regard.

Concomitantly, the reports paint a problematic picture of underrepresentation in sharp disagreement with the (normative) expectation that individuals of all stripes disperse equally into the various regions of the social space. On the federal level, for example, data from the German Sports Confederation (DSB) is used to highlight the finding that only 9% of members and only 4.7% of voluntary functionaries have a migration background, leading to the conclusion that “compared to the proportion of 20% in the population, people from migrant families are still substantially underrepresented, especially among voluntary functionaries” (Commissioner for Migration, Refugees, and Integration 2014, 192). Similarly, in the political realm, the federal reports point to the underrepresentation of persons with a migration background among public administrators in government agencies and of “Germans with a migration background...in parties and parliaments on all federal levels” (Commissioner for Migration, Refugees, and Integration, 2014, 120, 184). And in the cultural sector, “[migrants] are underrepresented in cultural life – among the audience as well as ‘on stage’ in artistic activities of their own” (Federal Government of Germany 2007, 132).

In accordance with the sociological ontology of mobility research, such unequal distributions of individual atoms in social space are thought to result from inhibitions or blockages. Where individuals are implicitly assumed to harbor essentially identical aspirations, external hindrances are seen to prevent a normal diffusion of individuals with a migration background.

Thus, the various integration reports are replete with references to barriers in all guises and shapes, blocking the otherwise ‘natural’ trajectories of persons with a migration background into all folds of the social fabric. For instance, on the very first pages of the National Integration Plan, the formal statement of the Federal Government includes the assertion that “[w]hether migrants can unfold their potentials is also a matter of the social circumstances and barriers they face” (Federal Government of Germany 2007, 13). Such barriers are identified for various institutional sectors throughout the document. Regarding the educational system, for instance, it is said that “for children with a migration background a decisive hurdle in their transition to secondary education lies in the mastery of the German language” (Federal Government of Germany 2007, 63). In the medical system, “informational, cultural, and communicative barriers stand in the way of [migrants’] taking advantage of offers and possibilities of preventive care and the promotion of wellbeing.” (Federal Government of Germany 2007, 99). Finally, in order to dismantle “financial, linguistic, and social barriers” in accessing cultural institutions, free entry for children and adolescents is discussed as a possibility which “can in turn motivate parents to visit also” (Federal Government of Germany 2007, 133). Such an imagery of “barriers” permeates integration reports on all other governmental levels as well.

With the culprit located in “society” and social structures, the responsibility for corrective measures almost inevitably falls to social policy. In many ways, stylizing the problem in this way procures a “jurisdiction” (Abbott 1988) for administrative bureaucrats. Thus, according to the way integration offices frame the issue, it is the government’s obligation to “dismantle the barriers” that inhibit what is implicitly taken as a universal trajectory and dispersion of individuals in society. Indeed, even other potential factors such as a specific habitus and culturally determined aspirations and inclinations are here implicitly redefined as familial and cultural “barriers” and thus construed as something external to the migrant’s ‘true essence’ (for this construal of the “cultural barrier” see Cloward and Ohlin 1960, 99–101).

The initiative of an “intercultural opening” of administrative institutions is indicative of such a shift in assigning the responsibility for integration. From the outset, the perspective of an intercultural opening has been intimately tied to the ontology of “access barriers” (see, for instance, Barwig and Hinz-Rommel 1995; Gaitanides 1996; 2001). Thus, in the official presentation of Westlanden’s program of intercultural opening, the low representation of persons with a migration background among municipal administrators is attributed to “access barriers,” as is the low utilization of administrative services by persons with a migration background (e.g., consultative services, cultural offers, health screenings etc.).

Westlanden’s program sponsors officers that are placed in municipal administrations throughout the state; they are responsible for the intercultural opening of the local bureaucracy. Such officers are socialized into the world view and vocabulary of intercultural opening during regular network meetings and retreats organized by the state’s integration department. These meetings discuss (though only vaguely define) aspects such as access barriers, intercultural competence, the phenomenon and practice of intercultural opening, all of which gain their ultimate plausibility from the statistical perspectives on over- and under-representation central to research on occupational attainment and social mobility.

This state program thus serves as an ideal case to explore how this particular sociological model of society is established and made visible in the broader world of administrative bureaucracies.

## The “Interculturalization” of Administrative Bureaucracies

In his seminal study “The Pasteurization of France,” Latour (1993) highlights how Louis Pasteur was able to bring people everywhere in France to account for, and act against, “microbes.” As Latour (1993, 38–39) argues, for this to happen Pasteurians had to insert themselves into virtually all social relations as ubiquitous “spokespersons of the microbe.” The presence of the microbe relied first and foremost on the presence of the Pasteurians and their devices, i.e., “the lectures, the demonstrations, the handbooks, the advice, the articles that they produced” (Latour 1993, 38).

I argue that a very similar perspective as the one employed by Latour is useful for making sense of what happens in the state program of intercultural opening. Just as Pasteurians altered the social ontology of France by spreading the laboratory practices needed to make the microbe “visible” via actor-networks, so are the state-sponsored officers for intercultural opening enacting an ontology of persons with a migration background, access barriers, intercultural competence, etc., by implementing various instruments and strategies that are likewise designed to make such entities “visible.” In fact, my fieldwork among these state-sponsored officers made quite apparent that “visibility” was an emic category and a crucial orientation of their work. Many of the strategies of intercultural opening which they discussed and presented in network meetings consisted in making various aspects of their work literally “visible.” I discuss this process in the following subsections. I show how officers of intercultural opening promoted and enacted a particular ontology of intercultural opening and integration by using such material tools as: statistics and surveys on the population of migrants in the community and among the administrative staff and/or those seeking services; trainings and HR interview guides on intercultural competence; integration monitoring reports; and inventories of integration services in the community.

### Creating an “Absence” of Persons with a Migration Background

In many ways, officers responsible for intercultural opening are on hostile grounds in local administrations. As the chief state administrator in charge of the program conceded, it can become quite uncomfortable in municipal bureaucracies: “You’ll get something along the lines of: ‘Why? Intercultural competence? We don’t need intercultural competence. We’re no bigots! In fact, we’re doing a great job, anyone is free to attend our services’...Or they’ll say bureaucracies are inherently neutral...Of course, they are not” (interview). The reaction of one local administrator was especially telling in this regard. As an officer for intercultural opening mentioned, this particular official responded to her efforts of intercultural opening with the statement: “I don’t need you to explain the world to me” (interview). Indeed, in many regards it *is* a particular world that these officers are assigned to impose on the bureaucrats in the municipal administration.

Thus, for the officers, a key element of legitimating themselves and their work in the municipality is creating the visibility of the very problem they are commissioned to “solve.” In essence, this means two things: (1) creating the visibility of persons with a migration background in the community and, (2) in a related move, creating the visibility of their *absence* in the local administration. Where officers have the resources, implementing *municipal integration monitoring reports* is a crucial component of this process of making persons with a migration background visible. And indeed, several officers have succeeded in establishing elaborate statistical reports on integration in their communities, or have at least resorted to official census data to ascertain the percentage of persons with a migration background in the community. As one officer put it, it is “important to make the demographic structure in the community visible” (fieldnotes).

However, the key operation in constructing a problem to which intercultural opening is the solution lies in highlighting the discrepancy between the proportion of persons with a migration background in the community and the proportion of persons with a migration background among the administrative staff and/or the citizens seeking administrative services.

For this purpose, officers generally aim to employ an *internal survey* on the representation of persons with a migration background in their local administrations. Some officers have also put forth great efforts in making the proportion of persons with a migration background visible among those seeking administrative services. The officer of one municipality, for instance, undertook extensive surveys among the various administrations of the different communities within the county. She and her collaborators conducted semi-structured interviews based on a detailed questionnaire, which in some instances was also sent out for self-completion. They inquired into the migration background of the workforce but also asked for estimates of the proportion of persons with migration background among those utilizing various administrative services such as consultations of different sorts or educational, cultural, and sports-related offers.

Through such technical means of surveys and statistical analysis, officers essentially create the visibility of an *absence*. A practical guide, issued by the state of Westlanden, on how to implement such surveys for the purpose of intercultural opening is very explicit about this: “In order to *create an awareness of such an underrepresentation*, many communities include a survey on the migration background of all personnel in the context of their processes of intercultural opening” (my emphasis).

Such procedures thus displace an ontology of a “plenum”—a perfectly complete administration staffed with administrators going about their official business—with something that is seen as fundamentally lacking: an administration void of a representative share of persons with a migration background. Just as the crescent moon can only be perceived as a *half-*moon through the virtual intuition of the *full* moon (see Sartre 1956), so does the administration only become interculturally “closed,” as it were, through the intuited notion of an administration proportionately staffed with people with and without a migration background. Officers create, or, at a minimum, reinforce a particular perspective on the administrative world as they situate the administration before the virtual horizon of an interculturally open, i.e., representative, bureaucracy.

### Making Access Barriers Visible

By way of constructing the administration as lacking, officers at the same time advance the ontology of the barrier as the fundamental entity responsible for the underrepresentation of persons with a migration background among personnel and citizens seeking services. Moreover, the previously mentioned questionnaire developed by one of the officers to assess the status of integration work in the various communities within the county contained not only questions on estimated proportions of persons with a migration background but also a question asking what has been done so far “to welcome newly arriving members of the community and keep down access barriers as far as possible” (cited from questionnaire). Interestingly, the officer responsible for the survey noted that “most of the time, people say that access barriers do not exist or that they do not see any” (interview). Evidently, for many administrators, the notion of “access barriers” is not a conventional way of parsing administrative realities. Inevitably, then, the work of officers involves promoting a particular “way of seeing,” and be it simply by means of continually exposing administrators to the discourse and vocabulary of intercultural opening. The general aim is to get administrators to ask themselves, as one officer put it, “what could hinder people to come to me?” (interview). An ontology of a “level” administration where access is unimpeded, and universally so, is thus displaced by an ontology of an administration rife with barriers that selectively block out a certain demographic among those seeking services.

Indeed, surveys are just one of the more fundamental devices of performing and enacting such an ontology in local administrations. In principle, all activities of intercultural opening are undertaken with the understanding that these measures help “dismantle access barriers” for persons with a migration background. As officers become established in local municipalities as a representative voice of persons with a migration background, they inevitably make the ontology of intercultural opening visible simply by making *themselves* visible. As one officer put it, “Everyone knows me as the lady who loves integration” (fieldnotes). Not surprisingly, then, strategies of, literally, “making oneself visible” and “positioning oneself as an expert on intercultural opening” (fieldnotes) were explicitly and copiously mentioned in the various group presentations during the network meetings where officers were discussing essential procedures of their work.

For officers, HR is deemed a crucial site for dismantling access barriers responsible for the underrepresentation of persons with a migration background among the administrative personnel as well as among citizens utilizing services. It is here that persons with a migration background can be specifically targeted for recruitment. And here “intercultural competence,” a crucial capacity in the process of dismantling “cultural barriers” between administrators and their clientele, can be made an explicit job requirement. Thus, officers generally try to insert themselves into the recruitment process in one way or another, with some even sitting in on job interviews. Their purpose here is getting HR personnel to “see” intercultural competence of job candidates as well as the barrier of intercultural incompetence.

### Making Intercultural Competence Visible

As noted, in the ontology of intercultural opening, intercultural competence is deemed a central factor for dismantling barriers, which, so one must conclude, are understood to lie to a considerable degree in the intercultural *in*competence of administrators. Again, the legitimacy of the officers substantially rests on the extent to which they succeed in convincing others that intercultural competence is actually a “thing.” An important steppingstone to intercultural opening is getting the HR department to name this skill as an explicit job requirement in their job listings. Trainings for the administrative staff in intercultural competence are also a staple of intercultural opening. This is usually delegated to professional coaches and trainers specializing in the field of intercultural communication. Indeed, it is safe to say that within the last couple of years, a whole cottage industry for such trainings has mushroomed, much propelled by the “refugee crisis” of 2015, which has given the topic of integration an additional boost (on the industry of sensitivity trainings in the US see Kelly and Dobbin 1998, 978).

I had the opportunity to review the detailed course materials of a renowned institute for intercultural trainings which was recommended by several officers. The content mostly focuses on a specific concept of culture as an internalized and taken-for-granted way of perceiving and acting that should be considered when dealing with others in one’s daily professional life (see Table 1).

**Table 1:** Schedule for a training by an institute specializing in “intercultural competence”

Schedule (day 1)		Schedule (day 2)	
9:00 am	Round of introductions	9:00 am	Stereotypes – when they help, when they hurt
9:45 am	Why culture?	9:30 am	Communication – Understanding what the other means
10:45 am	Coffee break	10:30 am	Coffee break
11:00 am	When cultural orientations collide	10:45 am	Conflict management and de-escalation
12:30 pm	Lunch break	12:30 pm	Lunch break
1:30 pm	Dealing with “foreigners” (The ladder of abstraction)	1:30 pm	Case work
3:00 pm	Coffee break	3:00 pm	Coffee break
3:15 pm	Cultural differences – How people “tick”	3:15 pm	Summary and evaluation
4:00 pm	End of day 1	4:00 pm	End of course

Interestingly, the course does largely without specifically operationalizing the well-worn term “intercultural competence.” In essence, the course as a whole is in itself an implicit operationalization of the concept. Whatever happens in these courses, what is transmitted, discussed, and finally taken away from them, can be seen to constitute intercultural competence. In a way, then, the mere arrangement for such trainings by the state-sponsored officers is a way of making intercultural competence visible as a “thing.” Much like the first pair part of an “adjacency pair” in a conversation, the setting of the course “establishes a ‘conditional relevance’ upon anything that occurs in the slot that follows; whatever comes to be said there will be inspected to see how it might serve as an answer” (Goffman 1981, 6; Schegloff 1968; Sacks 1973). In essence, the course is in itself a prompt to “see” intercultural competence in whatever is shown and discussed on these occasions. Intercultural competence will consequently come to be understood as knowing about culture in the sense in which culture is defined and discussed in such trainings.

Things are not as simple when it comes to intercultural competence as a skill that is to be identified in job interviews. Here, intercultural competence must be “seen” and distinguished from intercultural incompetence in the responses the applicant gives to specific questions or in the information that he provides in his curriculum vitae.

Many municipalities in and outside of Westlanden have consequently made attempts to operationalize the concept for the purpose of job interviews. In the context of the state program discussed here, several officers and state-level administrators have together devised a formal guide for identifying intercultural competence in such settings. As one officer put it in an interview, “with this instrument we want to enable those who make hiring decisions to [recognize] certain indicators for [intercultural] competence in the process of the job interview.” A state-administrator who was closely involved in the design of the instrument literally spoke of a “search pattern for the identification of intercultural competence” (fieldnotes).

The guide recommends devising, and gives examples for, specific questions, good and bad responses, and concrete observational anchors that indicate abstract skills associated with intercultural competence, such as sociability (e.g., “refers to all participants”) or flexibility (“can easily adapt to new situations in the interview”). The guide forms an instructional device that in effect rehearses activities of “membershipping” (Garfinkel 1967) potential utterances by the job applicant under the rubric of intercultural competence (or incompetence). It serves as an induction into a specific language game by such means as “dividing the domain of scrutiny by highlighting a figure against a ground, applying specific coding schemes for the constitution and interpretation of relevant events, etc.” (Goodwin 1994, 606). Through its detailed examples of interview questions and possible answers, it instills a “professional vision” (Goodwin 1994, 606) capable of identifying intercultural competence.

For instance, the guide suggests describing the following hypothetical scenario to the applicant and asking the following questions: “There are parents with a migration background who cannot make themselves properly understood or participate due to language difficulties. How would you include them in the work at your institution? What kinds of offers are feasible?”

The guide then presents the following “possible response of the applicant” indicating “intercultural competence”:

It is important to determine whether there is a communicative problem or some other type of miscommunication, for instance whether the system of day-care facilities needs explaining. In case of linguistic obstacles, one should include an interpreter if possible or offer information materials in multiple languages. Depending on what is planned, one could also use images or sketches, or technical means such as online translation devices. As a further step one could consider offering the parents opportunities for personal development, such as participating in parent-coffee-clubs or language courses.

As already becomes clear from the hypothetical response, intercultural competence is seen largely as a function of the degree to which the applicant himself has mastered the language of barriers and obstacles and is able to relate potential strategies of resolving the situation to the lowering of such barriers. This is confirmed by the “interpretation of the applicant’s answer with respect to intercultural competence” given by the guide, which furthermore offers observational anchors for the core competencies associated with intercultural competence such as empathy and reflectiveness:

The person is aware that *linguistic barriers* can *impede communication* and is able to identify them. His offering of different means of communication shows specialized knowledge in the field. The fact that he considered means of strengthening the individual resources of the parents shows an empathetic, “customer-oriented,” and reflective approach to other people [my emphasis].

Such examples, and the material device of the manual as a whole, serve as an inroad into the hermeneutic circle of interpreting observable bits of behavior in terms of the underlying construct of intercultural competence and interpreting the invisible construct of intercultural competence in terms of the observable bits of behavior. It is an exercise in the “documentary method of interpretation” (Garfinkel 1967). The guide serves as a template for “ad-hocing” practices (Garfinkel 1967) as it subsumes various answers under the dimensions of intercultural opening. Thereby, intercultural competence is made visible and accountable. It is made a “thing.”

### Making Integration Visible

Since the issue of intercultural opening is embedded in the broader and perhaps more seasoned issue of integration, much of the persuasiveness and currency of the former depends on the topicality, public awareness, and, indeed, the very palpability of the latter. Thus, in many regards, officers saw themselves responsible for making “integration” visible also. Officers who had successfully established statistical integration monitoring reports saw one of the fundamental benefits of this instrument precisely in this very process of making integration concrete. Since many statistical reports on education, employment, etc. were already available from the administration and together comprised much of the same data, additional benefits of integration monitoring reports were not immediately apparent, as one officer conceded. However, according to her, with this particular compendium “one paints a picture of what integration means per se. And especially for the public, you can show how these processes develop over the years. Improvements in educational status of the second and third generation can be visually seen at a glance...One has the synopsis and the association of integration with all that” (interview). Thus, these instruments and reports visualize the effects of a gradual dismantling of “access barriers.”

Furthermore, a crucial part of the officers’ assignment lies in “creating transparency” regarding the status quo of integration services offered by governmental and non-governmental organizations in the community. As the chief-administrator responsible for the program noted, even in municipalities where officers are unable to rely on much cooperation from their administration, they can at a minimum proceed with taking stock of integration-related offers and services in the area. Often such inventories form part of the municipal integration reports.

Such stock-taking has the manifest benefit of enabling administrators to direct migrants in need of particular integration services towards potential service providers in the community. It has, however, the added and more latent benefit of once again consolidating a world of integration work. As different services and organizations are drawn together under the explicit label of “integration,” including those that only tangentially or incidentally touch upon aspects deemed relevant to integration, a “field” of integration work is perhaps as much created as it is illuminated. Almost by fiat, such services are declared an element of a comprehensive effort towards integration. By being claimed in this very sense and, consequently, by their very existence, these services thus once again attest to the reality of intercultural opening and integration as they indeed inadvertently come to *materialize* and *objectify* the problem focus of access barriers for persons with a migration background.

What is more, as interviews indicate, the creation of such “transparencies” may indeed elicit field effects much in line with sociological field theory. They illuminate openings and niches for integration services in the sense of a “space of possibles” (e.g., Bourdieu 1996, 234–239). As one former representative of charitable organizations in the capital of Westlanden noted, service providers were looking at the publicized inventories of integration projects, “asking themselves, what do we have for possibilities, how do we fit in?” (interview). Indeed, in various interviews there were even several indications of competitiveness among such providers. Such dynamics, which presuppose a taken-for-granted ontology of integration work and specific investments in integration-related pursuits, themselves inevitably reinforce and perpetuate a reification of the integration concepts at the heart of the construction of such a “field.”

### Acts of Translation

In the previous sections, we have explored how officers make visible the ontology of intercultural opening in local administrations by relying on devices such as statistical analysis, surveys, manual guides, and inventories. But why should administrators lend themselves to the project of intercultural opening? How do officers succeed in enrolling and enlisting bureaucrats in this enterprise? How do they get them to invest in this particular ontology? Indeed, as noted above, administrators in the bureaucracy often resented and opposed the ontology of intercultural opening imposed on them. Many took umbrage at the suggestion that the structures in place, or even themselves, were discriminatory against persons with a migration background.

In essence, the stabilization of an ontology inhabited by entities such as persons with a migration background, intercultural opening, intercultural competence, etc. depends on building a network in which all the entities mutually stabilize one another. The success of this stabilization depends on enlisting administrators in the municipality as specific actors in this network. It depends on capturing their interest, or more precisely, on officers convincing the administrators what their interests (in this ontology) are (Latour 2012, 5). In this sense, it requires yet another act of construction. While these so-called “translations” (Callon and Latour 1981) figured prominently in early ANT, they have been somewhat neglected in ANT’s more recent iteration of studies on performativity and need to be brought “back in” (Beunza and Ferraro 2019).

As noted above, the personnel managers investigated by Dobbin (2009; Kelly and Dobbin 1998) won corporate employers for diversity management by appealing to business arguments. Similarly, in municipal bureaucracies of Westlanden a key strategy that most officers have come to rely on in winning over reluctant administrators is the repackaging of intercultural opening as something that would make the administrators’ work easier. Some have even tinkered with the title of intercultural trainings, relabeling them as “intercultural conflict management” or “efficient communication” while still providing the same contents and topics. As one officer put it, it is a way of selling their initiatives as efforts to smooth out transactions with clients and prevent “frictional losses” of the energy invested by local administrators (fieldnotes). Another officer likewise explained that administrators could be brought on board by stressing that intercultural communication can “facilitate organizational processes” (fieldnotes). As another officer noted, a more multilingual approach to bureaucratic interactions was “sold to [administrators] as a strategy for reducing their workload” (fieldnotes).

Such translations can be decisive for enlisting local administrators in the program, and hence the ontology, of intercultural opening. Through such methods, administrators are brought to “buy into” a world populated with “persons with a migration background” who face “access barriers” that will impede not only them but also the smooth operation of the bureaucracy unless “intercultural competence” is utilized in client-administrator interactions. In convincing administrators that they too serve to benefit from such a “thing” as intercultural opening, the very “thingness” of the latter is further consolidated. It is black-boxed as a taken-for-granted entity as administrators themselves are translated into actors that are well-advised to heed what officers are saying about the motives and needs of “persons with a migration background” and about “culture” and “interculturality.”

## Conclusion

In adding perspectives of science and technology studies to a literature on the construction of diversity management, this article has shown how officers for “intercultural opening” enact a sociological ontology of social mobility and structural barriers in municipal bureaucracies of the German welfare state. Officers assemble an actor-network, comprised of people and material devices, that channels specific templates for parsing reality into municipal administrations. Much like Latour’s (1993) account of Pasteurians, who, equipped with microscopes and petri-dishes, have inserted themselves as a third party into various social relations in order to “make room” for the microbe in the ontology of the social, officers in the program of intercultural opening, equipped with statistics, surveys, and manuals, insert themselves into local bureaucracies in order to reveal “absences” of “persons with a migration background,” “access barriers,” and “intercultural competence.”

In so doing, state-sponsored officers are first and foremost furnishing municipal administrators with a vocabulary and conceptual apparatus through which experience can be parsed in ways that resonate with and confirm the ontology of intercultural opening. These officers understand that this is a central component of their role. Several officers explained that they saw themselves primarily as “knowledge transmitters” regarding intercultural opening and as responsible for “sensitizing for its contents” (fieldnotes). They agreed that politically they could probably achieve very little in their position; instead, a fundamental goal for them was “that others in the administration will eventually have heard about such things [as intercultural opening etc.]” (fieldnotes). In precisely this regard, one officer was quite blunt on the merits of conducting two-day trainings on intercultural competence instead of one: two days were better because “things can be set” (fieldnotes). His peculiar choice of words betrays in an almost revelatory fashion what lies at the heart of the whole enterprise: constructing (“setting”) an ontology (“things”).

Essentially, then, officers aim to move administrators towards subsuming bits and pieces from the “blooming, buzzing confusion of reality” (James 1890) under the rubrics of intercultural opening. In many ways, “things” such as intercultural competence and integration must be seen in experiential data which could potentially be parsed and rubricated in myriad other ways, or not at all.

The last point is crucial. Much of the examples that the officers invoked for themselves as operationalizations of intercultural opening or intercultural competence could have been classified without reference to aspects of integration, migration background, or access barriers.

Indeed, during one of the network meetings, one of the officers more than once vented her frustration that people in her administration had unwittingly implemented a measure of intercultural opening without realizing that what they were doing “was” in fact a measure of intercultural opening. As she related, they had installed a job application procedure where applicants’ identities were anonymized but, to the officer’s exasperation, had ceased to list it under measures of intercultural opening during an external survey on the progress of intercultural opening in municipal administrations. In other words, they had not been able to see “intercultural opening” in a measure which to the officer was so clearly and unambiguously a measure of intercultural opening but had “simply” taken it as a fair-hiring initiative (fieldnotes).

As noted above, the inventorying activities of officers can similarly end up listing services as elements of the field of integration work which could have been seen, and indeed may see themselves, as much less “ambitious” instances of an ethnic organization merely providing some helpful services for members of their ethnic community or as general social programs, without necessarily foregrounding a broader agenda of “integration” and “diversity.” Once again, through such acts of parsing, which are more likely to catch on in the administration the more it is exposed to such concepts by the state-sponsored officers, an ontology of intercultural opening is reflexively confirmed in the very sense of ethnomethodological reflexivity. As such accounts of reality are continually indexed with the terminology of intercultural opening, administrators are urged towards seeing “intercultural-opening-in-the-talk,” to paraphrase the formula of seeing “society-in-the-talk” by Zimmerman and Pollner (1970, 92). It is about seeing “absences” of persons with a migration background where one had formerly seen the plenum of a normal administration; it is about seeing “intercultural competence” where one might just as well see simple instances of tact, courtesy, and patience; it is about seeing “integration work” where one could also see basic examples of intra-ethnic outreach or general social work; and it is about seeing “integration” where one might see simple measurements of educational attainment and employment. It is in this sense that the ontology of intercultural opening and diversity management is made “real.”

Indeed, in some instances, local administrators were quite aware that some of those activities that were now explicitly billed under integration had essentially been going on before. In one participating municipality of the state program for intercultural opening, a social worker and community organizer noted how what had previously been called “neighborhood management” had been relabeled as “integration management.” Yet, he said he and his team had simply continued with “their everyday work” (interview). He explained that since 65% of the people in his neighborhood had a migration background, they had effectively been doing integration work the whole time. The renaming simply opened up new sources of funding from the state. (In fact, the promise of such funding can be seen as another instance of translation, albeit one that did not involve much cunning and effort by the officers for intercultural opening).

These findings complement work which has pointed to the professional interests of personnel managers in installing practices of diversity management in organizations. They highlight the “ontological effort” implied in getting members of the organization to situate themselves in a self-evident world of diversity, access barriers, and intercultural competence. Enacting such a world depends on an infrastructure of sociotechnical devices. After all, the world of diversity management is a world made up of sociological “facts.” And like all scientific facts, they “are like trains, they do not work off their rails” (Latour [1983] 2012, 15). They have to travel into municipal administrations on the tracks of statistical devices, surveys, manuals, and inventories. It is through such sociotechnical assemblages that officers establish particular ways of seeing and knowing an otherwise indeterminate reality amenable to other modes of parsing (on this see also Daston 2008).

In the US, such sociotechnical assemblages were able to establish ideals of a proportionate representation of minority groups and a social-scientific ontology of structural barriers despite their contradiction with cultural legacies of color-blind liberal individualism. Similarly, in Germany, they have helped install the same ideals and ontologies even though issues of (anti-)discrimination, race, and ethnicity had largely been absent from public discourse before.

In showing how sociological models of the social world are established outside academia, this article also expands the recent turn of science and technology studies to the social sciences and the ways they intervene in, and shape, the world. While extant research has largely limited itself to the field of economics and the stabilization of market ontologies, this article broadens the scope in focusing on the enactment of *sociological* ontologies.

In fact, as this case shows, it largely falls upon such positions as officers for intercultural opening, diversity managers, and HR personnel – sociologists “in the wild,” as it were (see Callon 2007, 336)—to invest with reality many a concept devised in the ivory tower of academia and its intersections with policy discourse. People in such positions can in many ways be seen as the “foot soldiers” of performativity. They are struggling to enact the very reality of often obscure concepts such as access barriers, integration, intercultural opening, and intercultural competence—i.e., to make them visible, accountable, “tell-a-story-aboutable” (Garfinkel 1967). In many ways, then, they are the ones facing the burden of actually “doing” the “society” that has been devised elsewhere, most often in the offices of those who are afforded the time and technologies to venture comprehensive models and diagnoses of the social.

## Footnotes

1 For an analysis that more generally explores how municipal “diversity” departments give meaning to the term in their everyday practice of implementing diversity policies see Schiller (2016).

2 Indeed, in his discussion on the German response to the EU race directive of 2000, which required European states to implement legislation against direct and indirect discrimination, Joppke (2007, 265) notes the “absence of a culture of antidiscrimination” in Germany, which also did not escape the legislators at the time. It has, of course, much to do with Germany’s less substantial and consequential legacies of colonialism and slavery (as well as with its more muted recognition thereof).
